# A natural language processing based technique for sentiment analysis of college english corpus

**DOI:** 10.7717/peerj-cs.1235

**Published:** 2023-02-17

**Authors:** Jingjing Xu

**Affiliations:** The School of Humanities and International Education & Exchange, Anhui University of Chinese Medicine, Hefei, China

**Keywords:** Natural language, English corpus, Cluster analysis, TF-IDF

## Abstract

The college English corpus can help us better master English, but how to obtain the desired information from a large number of English corpus has become the focus of information technology. Based on the natural language processing (NLP) technology, a sentiment analysis model is built in this article. An improved term frequency–inverse document frequency (TF-IDF) algorithm is proposed in this article, where the weighted average method is used to determine the emotional value of each emotional word. The inspirational words are used to obtain the English corpus’s emotional tendency and emotional value. The results show that the model has high classification accuracy and operation efficiency when selecting feature words. Compared with the TF-IDF, the improved TF-IDF algorithm added the necessary information weight processing and word density weight processing to two new processing links, which can significantly improve the efficiency of college English learning.

## Introduction

The English corpus can help us understand its characteristics and also help us master some fixed expressions (Xu & Gao, 2020). In the previous Internet model, the channels for providing information to users are single. Now the Internet model has changed, the change in this process undoubtedly brings new opportunities for the Internet. There are a large number of English language materials on the Internet. A person cannot browse all the information on the Internet. How to help users obtain the information he wants has become the focus of information technology (Hasan & Farri, 2019). Currently, the research of college English natural language processing is still in its infancy, especially the lack of dynamic language analysis. Based on natural language processing (NLP), this article makes a dynamic analysis of the English corpus, which can significantly improve the efficiency of college English learning.

The NLP enables computers, mobile phones and other electronic devices to recognize and understand human languages, saving operating time and improving work efficiency. As an essential part of NLP, text classification has become one of the hotspots in academic research  (Fang, 2018; Girshick et al., 2015). Text classification technology is widely used in life, such as spam filtering, text sentiment analysis and topic detection on social platforms. In the face of massive data in the Internet world, data collection, processing and classification through artificial ways have been unable to meet users’ needs. Text classification technology can help people manage vast amounts of data efficiently and orderly but also help people excavate the hidden information and rules in the data. Text sentiment analysis is a branch of natural language processing that has shown good performance in microblog comments, news corpus and product evaluation (Fang, 2018).

The research on college English natural language processing is still in its infancy, and there is a lack of discussion on English emotion analysis. The dynamic analysis of college English corpus based on NLP can significantly improve learning efficiency. The rest of the article is organized as follows. The related work is introduced in the next section “Related work”. The construction of the sentiment analysis model of the college English corpus based on the improved TF-IDF model is discussed in the “Sentiment analysis model of college English corpus based on improved TF-IDF model” section and finally, the testing and the performance of the model from many aspects is discussed in “The experimental process” section.

## Related work

### Natural language processing technology

In recent years, NLP technology has been a research hotspot in the artificial intelligence industry. Two types of neural network models are primarily used: the convolutional neural network (CNN) and the cyclic neural network (Hu et al., 2016). The NLP is an essential direction in this field and is widely used in machine translation, public opinion monitoring and speech recognition (Liu, Zeng & Li, 2014; Luo et al., 2018). The earliest research work on NLP is machine translation; Weaver first proposed a machine translation design. The NLP technology mainly includes information extraction, automatic summarization, speech recognition technology, transformer model, NLP technology based on traditional machine learning and NLP technology based on deep learning (Xi & Zhou, 2016). NLP technology has been widely used in recent years, but some problems still limit its further development (Yin, Wang & Wang, 2015). According to common sense, the words between sentences are usually closely linked, and the meanings of words in different contexts are often different, which is easy to cause misunderstanding (Vinyals et al., 2015).

### Text sentiment analysis

Text sentiment analysis is a branch of natural language processing. The mainstream text sentiment analysis methods mainly focus on three categories: the dictionary-based, the rule-based and the machine learning methods (Yang, 2019; Abe & Tsumoto, 2010). The Internet generates many valuable comment information, which often contains people’s various emotions (Gui, 2018; Sun, 2019). The fusion of TextBlob and deep learning models also have tremendous applications in Twitter data sentiment analysis. By browsing these reviews, users can get their views on specific events. The text sentiment analysis is divided into four categories: keyword recognition, lexical association, statistical methods and concept-level technology. Keyword recognition is using clearly defined words to affect the classification to excavate opinions in a specific context. The relationship between grammar needs to be used (Mikolov et al., 2017; Li, Cao & Cao, 2018). The correlation between grammar often needs to be obtained through in-depth analysis of texts. The concept-level algorithm can detect subtle emotional expressions between words. There are many open-source software to calculate the sentiment analysis of large text sets, which include online news, online reviews and blogs (Silver et al., 2016).

## Sentiment Analysis Model of College English Corpus Based on Improved TF-IDF Model

Before applying the NLP technology to college English corpus sentiment analysis, the appropriate algorithm should be selected. The term frequency–inverse document frequency (TF-IDF) algorithm cannot assign different weights to words according to their location, so this article proposes an improved TF-IDF algorithm, which adds critical information weight processing and word density weight processing. The improved TF-IDF model can extract the sentiment of the college English corpus and effectively realize the sentiment analysis of the college English corpus, improving the accuracy of text classification.

### The overall framework of the model

**Figure 1:**
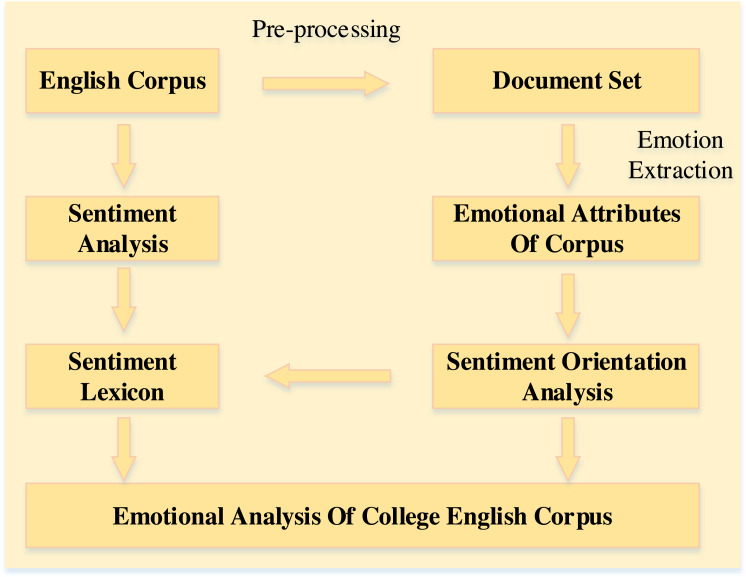
The overall framework.

The emotion analysis model aims to analyze the emotion of the college English corpus, which mainly includes data collection, preprocessing, emotion extraction and analysis. The overall framework of the model is shown in Fig. 1.

As shown in Fig. 1, the model starts from the data collection part and ends with the presentation of the interface. (1) The data are collected from the recent college English corpus through the crawler part and then saved into a document set of comments. The document set is used for preprocessing and constructing a general dictionary of sentiment orientation. (2) The text to be analyzed can be obtained from the preprocessed documents after part-of-speech tagging, dependency parsing and co-reference resolution. (3) The attribute information of the English corpus in the document needs to be extracted to obtain the attribute database. (4) The sentiment words are divided into attribute sentiment words and general sentiment words, which are applied to the sentiment analysis algorithm of the English corpus. Then the sentiment tendency and sentiment value of the English corpus are obtained.

### The data acquisition

The data used in this article are from the American Contemporary English Corpus, composed of one billion words of text. The text is divided into five categories: spoken language, novel, popular magazine, newspaper and academic journal. These five types of texts are evenly distributed. The COCA has a vital text retrieval function and divides the corpus in detail according to the year. The COCA focuses on the extensive information of nearly 60,000 words, including frequency information, definition, synonyms, *etc*. In addition to its incomparable advantages over other corpora, the Corpus of Contemporary American English also combines corpus and retrieval software, which can help language researchers analyze and study corpus conveniently and quickly. The American Contemporary English Corpus combines corpus and retrieval tools, and its online retrieval system can help users observe English vocabulary use.

### The improved TF-IDF mode

The TF-IDF algorithm can evaluate the importance of a word to its text, which can also realize the classification of text. The TF-IDF includes two parts (Zhou, 2019; Peng, 2019), the word frequency and the inverse text frequency, as shown in Formula (1).

(1) \begin{eqnarray*}\text{TF-IDF}=\mathrm{TF}\times \mathrm{IDF}.\end{eqnarray*}

By definition, the TF-IDF algorithm is the product of TF and IDF values, where TF is defined as Formula (2):

(2) \begin{eqnarray*}\mathrm{TF}= \frac{N({w}_{i},d)}{S} \end{eqnarray*}

where *N*(*w*_*i*_, *d*) represents the number of times a word appears in the text, *S* represents the total number of all words in the document.

The fewer the words, the smaller the TF value and the less important the words are to the text. When evaluating the specific ability of a word to the whole text set, it also needs to be judged by the IDF value. The definition of IDF is shown in Formula (3).

(3) \begin{eqnarray*}\mathrm{IDF}=\log \nolimits \left( \frac{N}{N({w}_{i})} \right) \end{eqnarray*}

where *n* represents the total amount of all text in the text set, *N*(*w*_*i*_) represents the total number of texts in which this word has appeared in the text set. The smaller the value of *N*(*w*_*i*_), the larger the IDF value, indicating that a word appears less frequently throughout the text set. The word will have a solid ability to distinguish categories.

**Figure 2:**
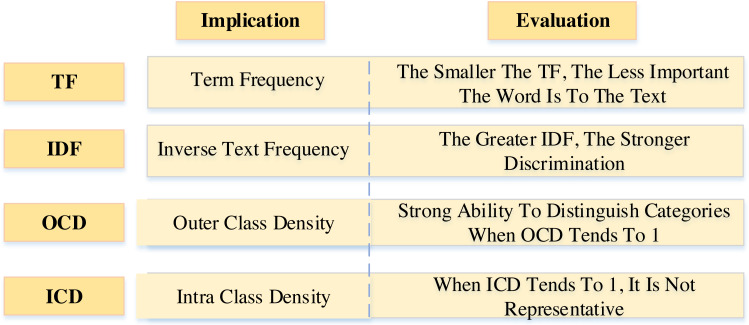
The main contents of the algorithm.

Suppose the probability of a word appearing in one text is very high, but the probability of its occurrence in other texts is very low. In that case, it can be considered that the word has a good classification ability and can be classified as a feature word. The TF-IDF algorithm believes that words with minor text frequency have more ability to distinguish text categories. In contrast, words with more significant text frequency are useless, which is not entirely correct. This article proposes an improved TF-IDF algorithm, which adds two new processing links: critical information weight processing and word density weight processing. The improved TF-IDF algorithm is very suitable for sentiment analysis of college English corpus, which mainly includes four parts, as shown in Fig. 2.

The frequency parameter of the occurrence of words is also introduced. The calculation formulas are shown in Formulas (4)–(5):

(4) \begin{eqnarray*}& & \mathrm{ICD}=\sqrt{ \frac{\sum _{k=1}^{n}(WF({w}_{i},{c}_{jk})- \frac{WF({w}_{i},{c}_{j})}{n} )^{2}}{n-1} }\end{eqnarray*}

(5) \begin{eqnarray*}& & \mathrm{OCD}=\sqrt{ \frac{\sum _{j=1}^{m}(WF({w}_{i},{c}_{j})- \frac{WF({w}_{i})}{m} )^{2}}{m-1} }\end{eqnarray*}

where *WF*(*w*_*i*_, *c*_*jk*_) represents the number of feature words, *WF*(*w*_*i*_) the total frequency of feature words in all text categories, and M represents the total number of text categories.

The value range of ICD is 0∼1. When the ICD value tends to 0, it shows that the model can well reflect the commonness of the text [20]. When the value tends to 1, the density of representative feature words in this text type is uneven, which cannot reasonably reflect the commonness of such texts. The threshold value used to calculate the similarity between features is 0.3.

The range of OCD values is the same as the ICD. When the value of OCD tends to 0, the words have a relatively average occurrence density in different text types, which cannot sufficiently represent a particular text type. When the value of OCD tends to 1, the density distribution of feature words in different categories is uneven.

When the ICD of a feature word tends to 0, and the OCD tends to 1, the word has a strong representation ability for a specific text type. The improved TF-IDF weight calculation function is finally formed and combined with ICD and OCD, as shown in Formula (6).

(6) \begin{eqnarray*}\text{Improved TF-IDF}=\mathrm{TF}\times \mathrm{IDF}\times \mathrm{OCD}\times (1-\mathrm{ICD}).\end{eqnarray*}

TF represents the word frequency, IDF represents the reverse text frequency, OCD represents the word density within the category, and ICD represents the word density outside the category.

### The emotion extraction of corpus

Before sentiment analysis of the English corpus, data cleaning should be done to eliminate the gap between data units. Data integration mainly integrates different data on data patterns and then detects and solves the conflict of data values. With data integration, you can discover and modify inconsistent data source naming and find and delete duplicate data in the data source.

Then, the emotional words are used to express the emotional tendency of users, most of which exist in adjectives, adverbs and nouns. In the emotional analysis, we need to obtain the polarity and intensity of each inspirational word. We hope to build a comprehensive sentiment database by referring to the English dictionary as much as possible.

**Figure 3:**
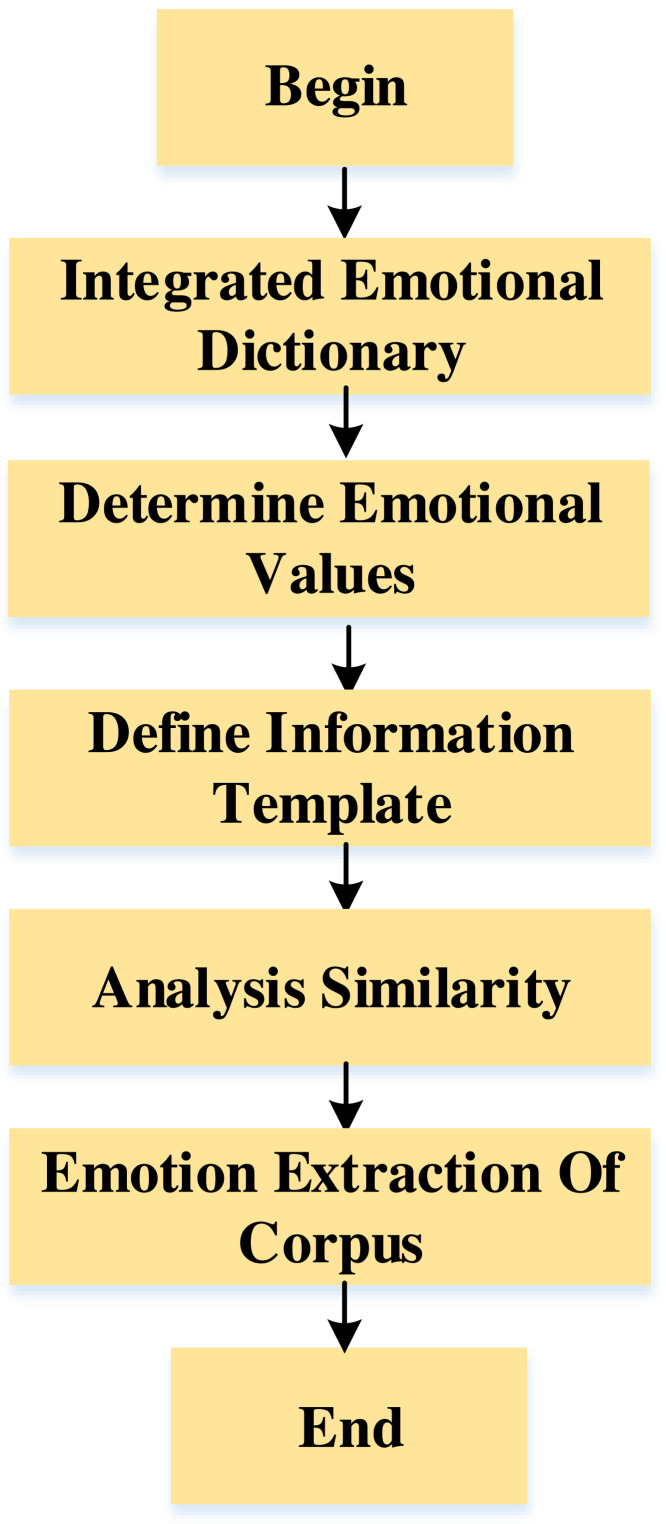
The emotion extraction of the corpus.

As shown in Fig. 3. In emotion analysis, the weighted average method is used to determine the emotional value of each inspirational word. When the emotional weight is more significant than zero, it indicates that the corpus tends to be positive. The closer the emotional value is to 1, the more obvious the positive tendency of the corpus is. When the emotional weight is less than zero, it indicates that the corpus tends to be negative. The closer the emotion value is to −1, the more obvious the negative tendency of the corpus is. Because the number of emotional words is too large, this article delimits the threshold range of emotional values, hoping to obtain words with more obvious polarity.

The effective attribute of the English corpus is the dimension to describe the emotion of the corpus, which is divided into two parts: the explicit attribute and the implicit attribute. The implicit attributes are difficult to extract and are not explicitly expressed in the statement but are hidden in the semantics. This article also defines an information template, the nouns obtained by the template are not all attribute words, which requires us to remove those false features. Therefore, confidence in the features is introduced, as shown in Formulas (7) and (8):

(7) \begin{eqnarray*}& & C= \frac{#PE(i)\times \sum _{j}{P}_{j}(i)}{N} \end{eqnarray*}

(8) \begin{eqnarray*}& & {P}_{j}(i)= \frac{{\mathrm{count}}_{j}(i)}{\sum _{t}{\mathrm{count}}_{j}(t)} \end{eqnarray*}

where *i* represents the feature number, *j* the pattern, the number of features consistent with, and the probability of feature *j* extracted through mode *i*.

According to the different dimensions of description, an English corpus can have multiple attributes, but these attributes are not independent. Some features can be divided into various features because of the different words. Their essence may belong to a more abstract concept. Therefore, it is necessary to divide the attribute database of the English corpus further. We will cluster English corpus attribute words to obtain deep-level English corpus attributes. The clustering algorithm can divide some words with similar or similar meanings into one category, highlighting the proprietary attributes of the English corpus.

The ML algorithm is not only easy to over-fit, but also has poor generalization ability. Therefore, this method is not selected in this article. The clustering algorithm can find the most similar features to the candidate features. If their similarity is greater than the threshold, they are considered to belong to the same category. The discriminant algorithm for noun phrases is relatively relaxed. If a noun phrase features belong to a particular category, that phrase belongs to this category. If this phrase does not contain noun features, it constitutes a separate classification.

The candidate feature set contains some non-truth features, so we first need to assign a value to each generated classification. With this method, the features with lower confidence may be preserved because they are classified into higher confidence classes. After the attribute words extraction and clustering algorithm, the attribute information of the English corpus is obtained.

### The emotional analysis of the corpus

After obtaining a large number of emotional words, we can sort out the polarity and emotional value of these emotional words. However, the polarity of these emotional words may not be consistent when they are used to describe different objects. Given the above phenomenon, we identify the emotional words with the polarity reversal phenomenon, the words with polarity reversal phenomenon are found out, and the attribute emotion dictionary is generated.

The attribute emotion dictionary is divided into three parts: the first part is the attribute characteristics, the second is the emotional words, and the third is the corresponding emotional value. It can be seen that the same emotional word has different emotional tendencies when describing other attributes, so the establishment of an attribute sentiment dictionary can effectively improve the accuracy of sentence orientation judgment. In the sentiment analysis of the English corpus, we follow the sentence structural stratification theory. An emotional clause of an attribute must contain one or more attributes of the product.

After determining the analysis principle, each evaluation sentence is cut into multiple clauses and the attribute evaluation words are obtained. With the analysis tool of Stanford, we can get the dependency relationship between sentence elements. Thus, we can get the formula of emotional analysis. We develop different algorithm strategies according to different modification relationships; different templates may share the same computing strategy.

## The Experimental Process

### The evaluation index

The college English corpus sentiment analysis has a complete evaluation system, which can measure the pros and cons of different information systems. The evaluation system helps to reflect the characteristics of other models, which can analyze the impact of various factors on the system. Precision and recall are often used as measurement indexes in information retrieval and statistical classification methods. The precision measures the accuracy of model classification or retrieval, as shown in Formula (9).

(9) \begin{eqnarray*}\text{Precision}= \frac{\mathrm{TP}}{\mathrm{TP}+\mathrm{FP}} .\end{eqnarray*}

When the actual results are positive, TP represents that the prediction results are correct. When the actual effect is negative, FP represents the correct prediction result.

The value of precision is between 0 and 1. The higher the accuracy is, the higher the proportion of correct retrieval is.

The recall measures the recall rate of the system, as shown in Formula (10).

(10) \begin{eqnarray*}\mathrm{Recall}= \frac{\mathrm{TP}}{\mathrm{TP}+\mathrm{FN}} .\end{eqnarray*}

When the actual results are positive, TP represents that the prediction results are correct. When the actual impact is positive, FN represents the wrong prediction result.

The value of the recall rate is limited between 0 and 1. The higher the value of the recall rate is, the less the model will miss when dealing with this category.

### The experimental process

The emotion analysis of the college English corpus is mainly divided into the following three steps. After obtaining the original data from the dataset, the sentiment analysis of the college English corpus should be carried out according to the following three steps. (1) Firstly, the data cleaning work should be completed. The emoticons and multiple landmarks in the data are removed to obtain the available experimental data. (2) The improved TF-IDF algorithm is used to extract the emotional features of the college English corpus. Each emotional word can correspond to an emotional polarity and an emotional value representing emotional intensity. (3) The emotional words are divided into attribute emotional words and general emotional words, which are applied to the analysis algorithm of sentence emotion to get the emotional tendency and emotional value of the English corpus.

## Results and Discussion

### The selection of feature words

**Figure 4:**
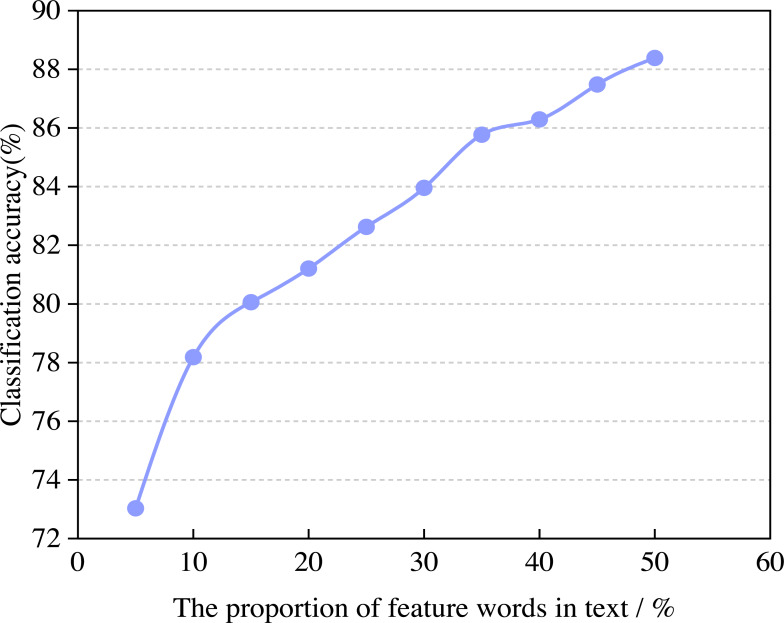
The classification accuracy of the model.

In the experimental process, the number of feature words has a specific impact on the accuracy and efficiency of the model. In this article, the change rule of classification accuracy and operational efficiency with the proportion of feature words is calculated through experiments. In the experiment, the training set and test set are set to 10,000 and the best proportion of feature words can be selected by adjusting the proportion of feature words, the number of iterations is 500. The variation of classification accuracy with the proportion of feature words is shown in Fig. 4.

From Fig. 4, we can see that when the proportion of feature words in the text is 5% and 50%, the classification accuracy can reach 73.03% and 88.39%. When the feature words account for more than 15% of the text, the classification effect of the algorithm tends to be gentle, and the classification accuracy reaches saturation. The reason is that when the proportion is small, the model can recognize the attribute and general sentiment words well. As the proportion continues to increase, more than half of the English corpus that should belong to attribute sentiment words will be wrongly assigned to general sentiment words. Similarly, the English corpus that should belong to general sentiment words will also be wrongly assigned to attribute sentiment words. Overall, with the increase in the proportion of feature words in the English corpus, the model’s classification accuracy gradually increases.

**Figure 5:**
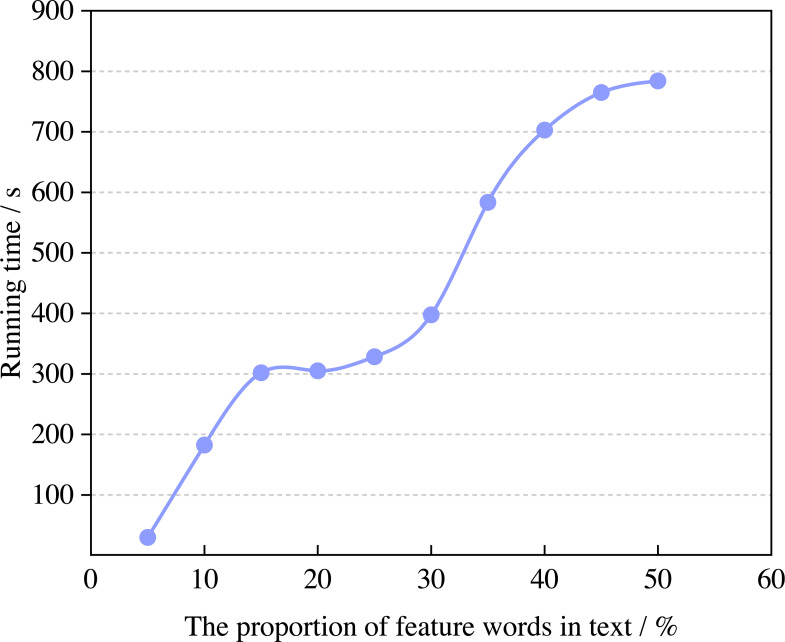
The running time of the algorithm.

Next, this article calculates the change rule of operating efficiency with the proportion of feature words, and the results are shown in Fig. 5.

According to Fig. 5, the running time of the model increases with the proportion of feature words and when it is 5% and 50%, the running time of the model can reach 29.83s and 784.00s. When the proportion in the text is 5%∼30%, the running time of the model tends to be saturated. The emotional attribute words in the English corpus have high similarity. The attribute words cost, price and payment have similar meanings in the attribute of price, which belongs to the deep attribute of price. With the increase in the proportion of feature words in the English corpus, the increasing workload of model recognition will naturally increase the running time, which is also consistent with common sense.

### The precision of the model

**Figure 6:**
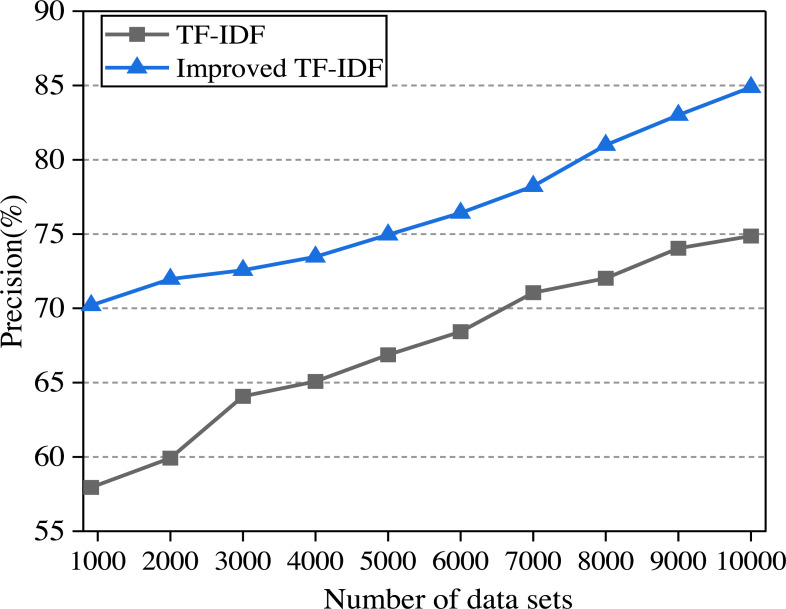
The comparison of algorithm precision.

In this article, the two algorithms are compared and the experimental results are shown in Fig. 6. In the experiment, the college English corpus is sorted from small to large according to the number of texts. With the increase in the number of training sets, the performance of different algorithms for text classification accuracy is observed.

It can be seen from Fig. 6 that the precision of the improved TF-IDF algorithm is higher than that of the traditional TF-IDF. With the increase of training set text, the precision of each classification model is also increasing. When the number of data sets reaches the maximum, the precision of TF-IDF and the improved TF-IDF algorithm is 74.87% and 84.88%, respectively. When the English corpus consists of two clauses, each clause describes one attribute of the English corpus, so we divide the sentence into two parts according to the clause. After getting the emotional values of the two clauses, we finally get the emotional analysis results of the whole sentence by weighting. Since the improved TF-IDF algorithm increases the weight preprocessing and word density processing, the precision can be increased by 7.38%∼23.83% compared with the traditional algorithm.

### The recall of the model

**Figure 7:**
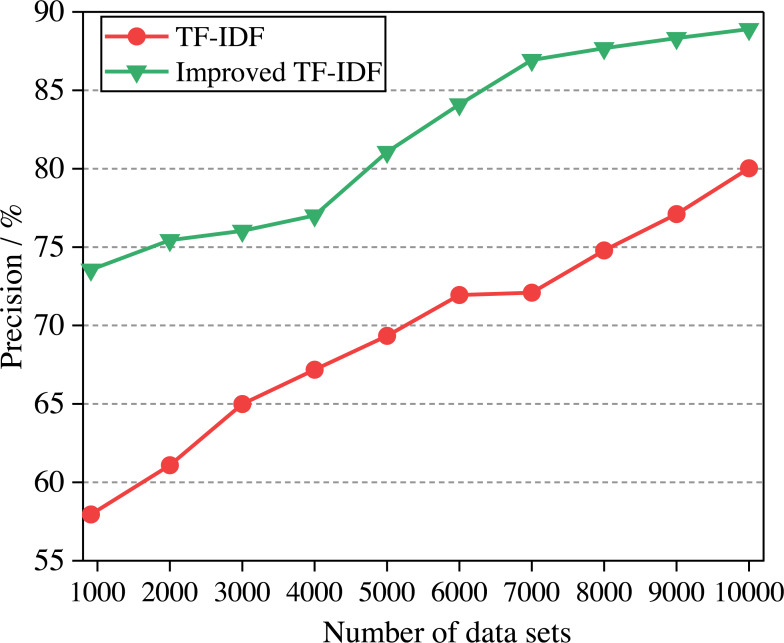
The comparison of algorithm recall.

Control experiments with different data volumes are also carried out to evaluate the algorithm’s recall. The two algorithms are used for experiments, and the experimental results are shown in Fig. 7.

 It can be seen that the recall of improved TF-IDF is higher than that of the TF-IDF algorithm. The recall of the model increases with the increase of data sets, and the recall rises linearly with the number of data sets. The emotional polarity of emotional words may not be consistent for different description objects. When ‘high’ is used to describe prices, it means that prices are too high, which is negative. When it is used to describe quality, it means high quality, which is positive. Therefore, when the number of data sets reaches the maximum, the recall of TF-IDF and improved TF-IDF are 80.027% and 88.900%, respectively. The recall of the improved TF-IDF algorithm is increased by 8.19%∼26.84%.

## Conclusion

The sentiment analysis model of college English corpus based on NLP technology is established in this article, in which the weighted average method is used to determine the emotional value of each emotional word. The emotional words are divided into attribute emotional words and general emotional words, which are then applied to the analysis algorithm to obtain the English corpus’s emotional tendency and emotional value. Then, the sentiment analysis model is introduced in detail from four aspects: the overall framework of the model, the improved TF-IDF model, the sentiment extraction of the college English corpus and the sentiment analysis of the college English corpus. Finally, the model’s performance is tested. The results show that the improved TF-IDF has a high classification accuracy of feature words and high operation efficiency. Compared with the TF-IDF, the precision and recall of the model have been greatly improved, which can greatly improve the efficiency of college English learning. Compared with the traditional algorithm, the improved TF-IDF algorithm has great application potential in feature word classification, operation efficiency and accuracy. Although the model proposed in this article has good performance, the data set used in this article is limited, which needs further research. This article only studies the performance of the improved TF-IDF algorithm before and after improvement. Next, this article needs to compare with more methods. We will work more on the novelty they are proposing over current SoTA methods rather than TF-IDF which already lacks in complex tasks such as sentiment analysis.

##  Supplemental Information

10.7717/peerj-cs.1235/supp-1 Supplemental Information 1 Code used to implement the technique

## Additional Information and Declarations

### Data Availability

The following information was supplied regarding data availability: The code is available in the Supplemental Files. The English corpus dataset is available at: https://www.english-corpora.org/.
